# Functional Mapping of Protein-Protein Interactions in an Enzyme Complex by Directed Evolution

**DOI:** 10.1371/journal.pone.0116234

**Published:** 2014-12-31

**Authors:** Kathrin Roderer, Martin Neuenschwander, Giosiana Codoni, Severin Sasso, Marianne Gamper, Peter Kast

**Affiliations:** Laboratory of Organic Chemistry, ETH Zurich, CH–8093, Zurich, Switzerland; Indian Institute of Science, India

## Abstract

The shikimate pathway enzyme chorismate mutase converts chorismate into prephenate, a precursor of Tyr and Phe. The intracellular chorismate mutase (MtCM) of *Mycobacterium tuberculosis* is poorly active on its own, but becomes >100-fold more efficient upon formation of a complex with the first enzyme of the shikimate pathway, 3-deoxy-d-*arabino*-heptulosonate-7-phosphate synthase (MtDS). The crystal structure of the enzyme complex revealed involvement of C-terminal MtCM residues with the MtDS interface. Here we employed evolutionary strategies to probe the tolerance to substitution of the C-terminal MtCM residues from positions 84–90. Variants with randomized positions were subjected to stringent selection *in vivo* requiring productive interactions with MtDS for survival. Sequence patterns identified in active library members coincide with residue conservation in natural chorismate mutases of the AroQ_δ_ subclass to which MtCM belongs. An Arg-Gly dyad at positions 85 and 86, invariant in AroQ_δ_ sequences, was intolerant to mutation, whereas Leu88 and Gly89 exhibited a preference for small and hydrophobic residues in functional MtCM-MtDS complexes. In the absence of MtDS, selection under relaxed conditions identifies positions 84–86 as MtCM integrity determinants, suggesting that the more C-terminal residues function in the activation by MtDS. Several MtCM variants, purified using a novel plasmid-based T7 RNA polymerase gene expression system, showed that a diminished ability to physically interact with MtDS correlates with reduced activatability and feedback regulatory control by Tyr and Phe. Mapping critical protein-protein interaction sites by evolutionary strategies may pinpoint promising targets for drugs that interfere with the activity of protein complexes.

## Introduction

**Figure 1:**
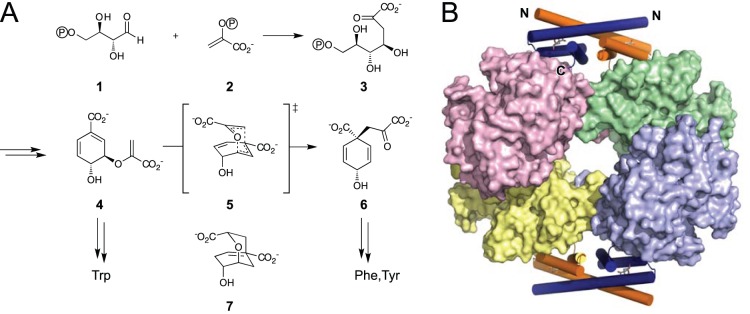
DAHP synthase and chorismate mutase reactions and structure of the MtCM-MtDS complex. (A) The shikimate pathway starts out with the condensation of d-erythrose-4-phosphate **1** and phospho*enol*pyruvate **2** to form d-*arabino*-heptulosonate-7-phosphate (DAHP) **3** catalyzed by DAHP synthase. DAHP is processed in six further enzymatic steps to chorismate **4**. Chorismate mutase (CM) catalyzes the pericyclic Claisen rearrangement from **4** *via* the presumed bicyclic transition state **5** to prephenate **6** [46]. A mimic of this transition state, Bartlett’s *endo*-oxabicyclic dicarboxylic acid transition state analog **7** is the best known inhibitor of most CMs [47]. (B) Hetero-octameric complex between MtCM and MtDS. MtDS forms a tetrameric core (shown in surface representation) which is flanked by two MtCM dimers (cartoon mode with α-helices represented as cylinders featuring **7** as a stick model with grey carbons in the active sites) that clamp the MtDS tetramerization interface (PDB: 2W1A) [10].

In prokaryotes, fungi, algae, and plants, the aromatic amino acids l-phenylalanine (Phe, F), l-tyrosine (Tyr, Y), and l-tryptophan (Trp, W) are biosynthesized via the shikimate pathway [1]–[3]. The initial step is the condensation of d-erythrose-4-phosphate **1** and phospho*enol*pyruvate **2** to 3-deoxy-d-*arabino*-heptulosonate-7-phosphate (DAHP; **3**) by DAHP synthase (Fig. 1A). Another six enzymatic steps afford chorismate **4**, which is the substrate for anthranilate synthase in the branch towards Trp biosynthesis, or which is converted by chorismate mutase (CM) to prephenate **6**, the precursor of Phe and Tyr. Feedback inhibition of strategically positioned enzymatic steps is a commonly used strategy in the shikimate pathway but it is implemented in different ways for various organisms [1]. For example, *Escherichia coli* produces three isoforms of DAHP synthase, each of them being sensitive to Tyr, Phe, or Trp [2]. In addition, the bifunctional CM-prephenate dehydratase [4], [5] and CM-prephenate dehydrogenase [6], [7] are inhibited by the products Phe and Tyr of the respective metabolic branches in *E. coli* [2].

In contrast, *Mycobacterium tuberculosis* possesses just one DAHP synthase (MtDS) and one intracellular, monofunctional chorismate mutase (MtCM). Whereas MtDS alone is strongly inhibited by the simultaneous binding of the three aromatic amino acids [8], [9], kinetic investigations have shown that the CM activity of MtCM itself is not subject to feedback control [10]. However, MtCM becomes sensitive to synergistic inhibition by Tyr and Phe upon formation of a non-covalent enzyme complex with MtDS. Structural characterization by x-ray crystallography has revealed that the complex consists of two MtCM dimers, which decorate a tetrameric MtDS core (Fig. 1B) [10]. The CMs from this hetero-octameric complex exhibit structural features that significantly deviate from the prototypic bacterial CM domain, the AroQ_α_ fold, as well as from the eukaryotic (AroQ_β_) and the secreted (AroQ_γ_) CMs, and were thus grouped as the AroQ_δ_ subclass [11], [12]. MtCM is the first AroQ_δ_ representative that was investigated in structural detail [10], [13]. It is assumed that the CMs of the shikimate pathway in the bacterial order *Actinomycetales*, which includes corynebacteria and mycobacteria, generally belong to the AroQ_δ_ subclass and are involved in a similar enzyme complex with the corresponding DAHP synthases [10], [14], [15].

**Figure 2:**
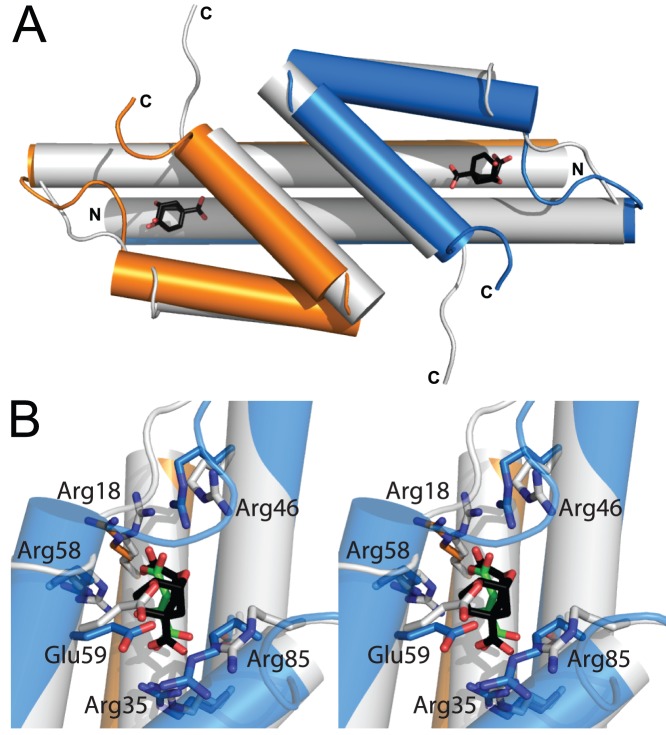
Comparison of free and MtDS-complexed MtCM. Superimposition in cartoon mode of free MtCM (white, PDB: 2VKL, active site liganded with malate) and MtCM from the complex with MtDS (MtCM subunits in blue/orange, PDB: 2W1A, active site liganded with **7** shown as sticks with black carbons). MtDS is omitted for clarity. (A) Overview with labeled N and C termini. Only the active site ligand of MtCM in PDB: 2W1A, the transition state analog **7**, is shown. (B) Wall-eyed stereogram of the superimposed active sites, with both liganded malate (sticks with green carbons) and **7**. Side chains of some active site residues, which change location upon complex formation, are shown as sticks [10].

Without MtDS, MtCM activity is reduced by two orders of magnitude. From the crystal structures of free MtCM and the MtCM-MtDS complex it is clear that MtDS residues do not directly participate in the acceleration of the chorismate to prephenate rearrangement [10]. Instead, it was speculated that the interaction with MtDS optimally positions MtCM active site residues for catalysis. Thus, the stimulation of CM activity must be indirect through transmission of conformational changes at the subunit interface to the catalytic center of MtCM (Fig. 2) [10]. However, it is poorly understood how individual protein segments participate in the more than 100-fold enhancement in catalytic activity. Some C-terminal positions of MtCM were previously probed by site-directed mutagenesis for their contribution to the activation mechanism. Whereas MtCM variants Arg87Ala, Leu88Ala, and a variant with a truncation before Leu88 showed catalytic parameters similar to wild-type MtCM in the absence of MtDS, activation upon complex formation was affected over a broad range of between only 2-fold (Arg87Ala) and 70-fold (Leu88 truncation) [10]. This finding suggested that the tested C-terminal residues are not involved in the basic catalytic machinery, but in the activation mediated by MtDS. To more systematically probe the activation mechanism, we implemented here an evolutionary strategy for the identification of patterns of MtCM residues compatible with formation of a productive complex with MtDS.

## Results and Discussion

### Rationale for randomizing MtCM residues

We chose the method of directed evolution [12], [16]–[18] to investigate the role of the C-terminal residues of MtCM in the formation of the complex with MtDS. Thereby, several gene libraries encoding MtCM variants with randomized C-terminal positions were generated and subjected to selection for CM function.

**Figure 3:**
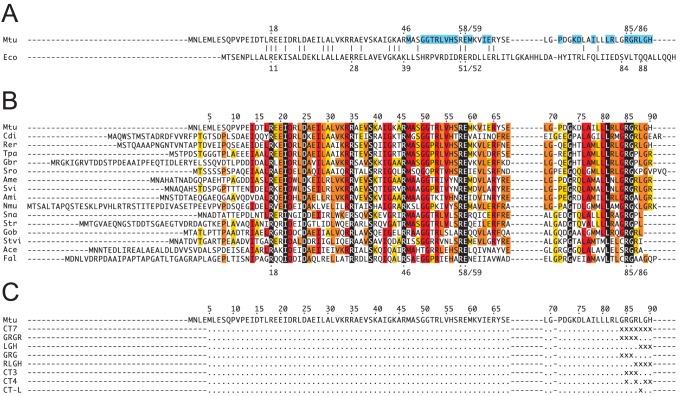
Sequence alignments of relevant AroQ chorismate mutases. (A) Structural alignment based on an overlay of X-ray structures of EcCM (PDB: 1ECM) and MtCM (PDB: 2W1A) [10]. Catalytic residues are indicated with dots and numbers above or below the primary sequence. Residues that could assume the roles of EcCM’s Ser84 and Gln88 are missing in MtCM. MtCM residues within a 6-Å shell of MtDS are highlighted in cyan. (B) Multiple sequence alignment of representative AroQ_δ_ CMs from the order of *Actinomycetales*. The conservation of individual residues is color-coded by text highlighting in black, as 100%; red, ≥75%; orange, ≥50%; yellow, ≥33%; white, <33% identity; numbering according to the MtCM (Mtu) sequence. Abbreviations: Mtu, *M. tuberculosis*; Eco, *E. coli*; Cdi, *Corynebacterium diphtheriae*; Rer, *Rhodococcus erythropolis*; Tpa, *Tsukamurella paurometabola*; Gbr, *Gordonia bronchialis*; Sro, *Segniliparus rotundus*; Ame, *Amycolatopsis mediterranei*; Svi, *Saccharomonospora viridis*; Ami, *Actinosynnema mirum*; Nmu, *Nakamurella multipartita*; Sna, *Stackebrandtia nassauensis*; Str, *Salinispora tropica*; Gob, *Geodermatophilus obscurus*; Stvi, *Streptomyces viridochromogenes*; Ace, *Acidothermus cellulolyticus*; Fal, *Frankia alni*. (C) Up to seven C-terminal residues of MtCM were randomized in libraries CT7, GRGR, LGH, GRG, RLGH, CT3, CT4, and CT-L. Randomized positions are indicated as “x”, wild-type residues as dots.

From superimpositions of the crystal structures of free MtCM (PDB: 2VKL) [10], MtCM in complex with MtDS (PDB: 2W1A) [10], and EcCM (the CM domain of the CM-prephenate dehydratase from *E. coli*; PDB: 1ECM) [19], and from an alignment of AroQ sequences, we identified several residues in the C-terminal region of MtCM as candidates for facilitating the boost in catalytic activity upon complex formation. While not contributing functional groups to the active site, the seven most C-terminal residues (positions 84–90) looked promising in this regard as they fulfill the following criteria: *(a)* they are proximal to MtDS in the MtCM-MtDS complex and are also in contact with active site residues. The six last residues are within 6 Å of MtDS (PDB: 2W1A; Fig. 3A; [10]), contacting ten out of the 20 MtDS residues at the interaction interface between the two enzymes. Arg85 additionally makes van der Waals contacts to ligand **7**, which is bound at the active site of MtCM. *(b)* They show structural and chemical dissimilarities to the prototypical AroQ_α_ protein, EcCM, which does not rely on activation by a complex partner. EcCM has two residues in the C-terminal portion of helix 3, Ser84 and Gln88, that are crucial for its activity. Upon Gln88Ala mutation, EcCM activity drops by a factor of 2×10^4^, and Ser84 is considered important for orientation of the substrate molecule in the active site [19], [20]. Both residues are missing in MtCM, and there are no chemically plausible substitutes in the C-terminal region (Fig. 3A). Furthermore, instead of an extended helix in EcCM, the C-terminus in MtCM adopts a loop structure. Upon complex formation, this loop rearranges such that the penultimate C-terminal residue changes its position by more than 14 Å [10]. Gly84 and Gly86 of MtCM may thereby serve as helix-breakers, allowing the C-terminus to bend away from the active site (Fig. 2A). *(c)* They show good conservation in the AroQ_δ_ alignment (Fig. 3B). In fact, the multiple sequence alignment of different AroQ_δ_ CMs exhibits a fully conserved Arg-Gly dyad at MtCM positions 85–86 and strongly conserved residues next to this pattern that are not found in AroQ_α_ proteins like EcCM (Fig. 3A).

### Redesign and calibration of the selection system for directed evolution

**Figure 4:**
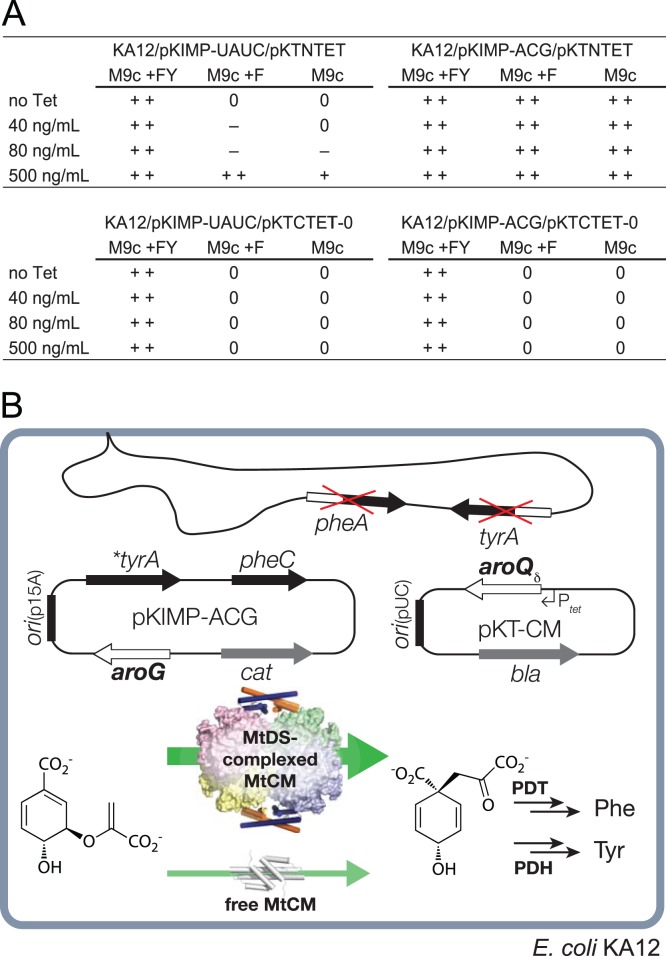
Selection system for MtCM variants that can be activated by MtDS. The selection system used is based on *E. coli* strain KA12 lacking the endogenous *pheA* and *tyrA* genes, which encode CM-prephenate dehydratase (PDT) and CM-prephenate dehydrogenase (PDH), respectively. Plasmid pKIMP-UAUC has the p15A-derived origin of replication (*ori*_p15A_) and carries *tyrA for a monofunctional PDH and *pheC* for a monofunctional PDT, in addition to *cat* providing chloramphenicol resistance [21]. Plasmid pKIMP-ACG additionally contains the *aroG* gene encoding MtDS. (A) Performance of wild-type MtCM (encoded by *aroQ_δ_* on plasmid pKTNTET) without MtDS (pKIMP-UAUC) or with MtDS (pKIMP-ACG) in comparison to a negative control (pKTCTET-0, lacking *aroQ_δ_*). Growth was assessed on M9c-based minimal plates in the presence or absence of Phe (F) and Tyr (Y). Colony size was scored either as++(good growth),+(moderate growth), – (poor growth), or 0 (no trace of growth) as a function of the added Tet concentration, the inducer of the P*_tet_* promoter upstream of *aroQ_δ_*. (B) Schematic representation of the redesigned selection system. The *aroQ_δ_* gene encoding an MtCM library variant is provided on plasmid pKT-CM that has otherwise the same structure as pKTNTET (*bla*, β-lactamase for ampicillin resistance; its *ori*_pUC_ is compatible with *ori*_p15A_). Under stringent selection conditions (M9c), host cells transformed with both pKIMP-ACG and a pKT-CM library plasmid can only produce enough prephenate and consequently Phe and Tyr needed for growth if the encoded MtCM variant can engage in a productive complex with MtDS (wide green arrow). Transformants having insufficient CM activity (thin, light green arrow) require exogenously added F and Y for growing on M9c minimal plates.

To select for MtCM variants that are still capable of productive interactions with MtDS, a previously established selection system [21] based on the CM-deficient and thus Phe and Tyr auxotrophic *E. coli* strain KA12 was adapted. KA12 carries a chromosomal deletion of the genes *pheA* and *tyrA* encoding the two bifunctional enzymes CM-prephenate dehydratase and CM-prephenate dehydrogenase, respectively. It can grow on minimal medium devoid of Phe and Tyr (M9c), if provided with the helper plasmid pKIMP-UAUC carrying the genes *pheC* and *tyrA for monofunctional versions of prephenate dehydratase and prephenate dehydrogenase, respectively [21], and, additionally, with a compatible plasmid containing a sufficiently active CM gene. Fig. 4A shows that the wild-type MtCM gene on plasmid pKTNTET complements the CM deficiency of KA12/pKIMP-UAUC on selective minimal plates. However, growth is only possible, if MtCM gene expression is induced with an elevated concentration (500 ng/mL) of tetracycline (Tet), the inducer of the P*_tet_* promoter upstream of the CM gene [17], [22]. In the absence of inducer or at lower Tet levels, growth is impossible or severely impaired. As an alternative to varying the Tet concentration for rigorous control of the intracellular enzyme level [17], [22], the stringency of the selection system can be tuned by providing Phe in the selective minimal medium (*i.e.*, M9c +F; Fig. 4A), such that the cells only need to biosynthesize Tyr for growth [21].

Fig. 4B illustrates an extended version of the selection system to explore MtCM sequence features important for activation by MtDS. Instead of plasmids pKIMP-UAUC and pKTNTET, KA12 contains, respectively, pKIMP-ACG, which additionally carries *aroG* encoding MtDS, and the library plasmid pKT-CM, which encodes partially randomized MtCM variants. Since pKT-CM has an otherwise identical structure to pKTNTET, expression of the *aroQ*_δ_ mutant genes is also controlled from P*_tet_*.

A possible concern is that endogenous *E. coli* DAHP synthases might affect MtCM activity. However, this is highly unlikely, since MtCM is a member of the distinct AroQ_δ_ subclass not present in *E. coli*, and the endogenous DAHP synthase isoenzymes, which belong to a different subtype than MtDS, are not part of a feedback regulatory circuit for controlling CM activity [10]. The lack of regulatory interactions between MtCM and the DAHP synthases of KA12 is also apparent in Fig. 4A where addition of Phe to M9c promotes –rather than feedback inhibits– growth of KA12/pKIMP-UAUC/pKTNTET.

The new KA12/pKIMP-ACG selection system was calibrated using wild-type MtCM (on pKTNTET) and appropriate negative controls. It was demonstrated (Fig. 4A) that at basal promoter levels (no or only little Tet added), survival of bacteria on selective minimal medium agar plates (M9c, M9c +F) depends on the formation of an active MtCM-MtDS complex (*i.e.*, the presence of both pKIMP-ACG and pKTNTET). These results showed that KA12/pKIMP-ACG can be used as a new selection system to explore the interactions between the two enzymes.

### Sequence patterns selected from libraries of C-terminally randomized MtCM

**Figure 5:**
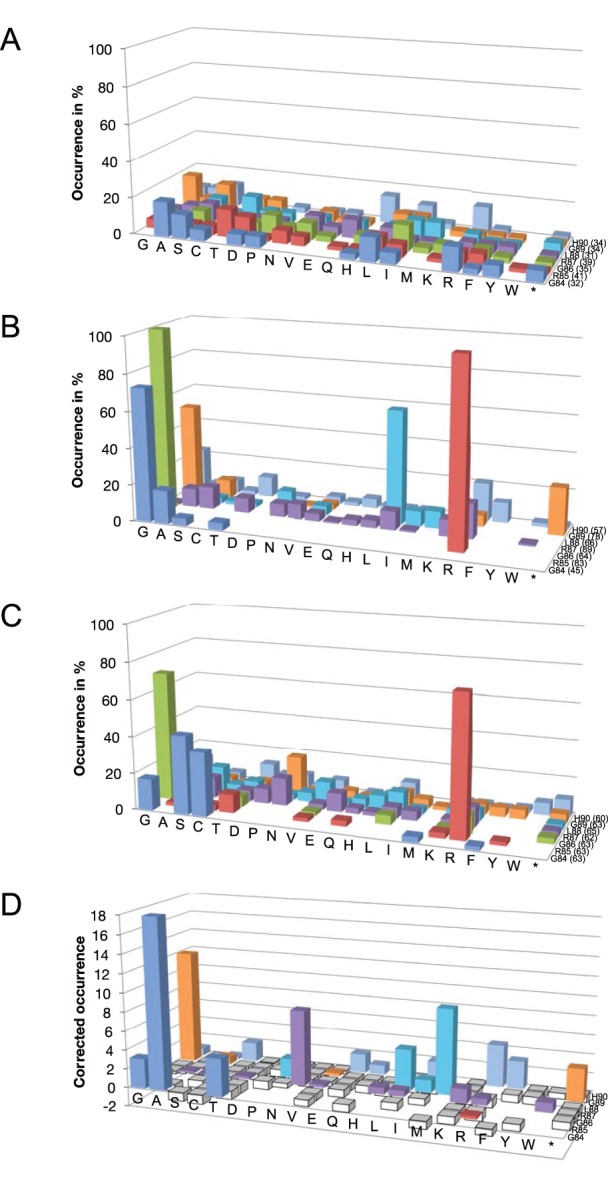
Amino acid distribution patterns in MtCM variants before and after selection experiments. Column colors correspond to the randomized positions 84 (blue), 85 (red), 86 (green), 87 (purple), 88 (cyan), 89 (orange), and 90 (light blue). Side chains are ordered according to increasing volume [48]; an asterisk denotes a stop codon. The absolute number of codons compiled at each position is indicated in parentheses next to the wild-type residue. The absolute numbers of individual residues found at every position are, in addition to the graphical representation of the relative frequencies shown here, listed in S1 Table. (A) Amino acid residues found under non-selective conditions (M9c +FY). The percentages (and standard deviations σ_n-1_) of the 4 individual nucleotides averaged over every randomized position were 25.9 (±7.3)%, 26.5 (±7.5)%, 21.6 (±6.3)%, and 26.0 (±6.0)% for A, C, G, and T, respectively, in the analyzed sample set. (B) Preferred residues selected under a stringent regime (M9c, no inducer added) in the presence of the complex partner MtDS (in KA12/pKIMP-ACG). (C) Residue patterns emerging in selected MtCM variants grown in the absence of MtDS under conditions where free wild-type MtCM can complement the CM deficiency (in KA12/pKIMP-UAUC, plated on M9c +F +Tet^500 ng/mL^). (D) Correlated amino acid distribution patterns. Positive values indicate a residue preference in the complexed MtCM, whereas negative values (columns in white) show residues frequently observed in functional free MtCM. To simplify the arbitrary graphical summary, and to avoid division by 0, the value at each position represents the ratio of [number of a particular amino acid found in complexed MtCMs +1]/[number of the same amino acid found in the free MtCMs +1] −1.

Eight MtCM gene libraries were constructed by randomizing different subsets of the codons for the seven C-terminal residues (Fig. 3C). This was accomplished with partially degenerate oligonucleotides encoding the mutagenized positions in the NNN codon format, deliberately including the three stop codons in the randomizing cassettes. The library plasmid pools were transformed into KA12/pKIMP-ACG and the transformants were plated on minimal medium agar plates. Library sizes determined from control platings on non-selective M9c +FY (supplemented with both Phe and Tyr) ranged from 3×10^4^ to 1.8×10^6^ clones per library. Sequencing of 65 clones from non-selective plates confirmed that the chemical oligonucleotide synthesis and the procedures used in the construction of the libraries did not inappropriately bias the composition of the libraries at the nucleotide level, which would have skewed the ensemble of amino acid sequences available for selection (Fig. 5A). The KA12/pKIMP-ACG libraries were subjected to selection on minimal M9c agar plates. After 3 days of growth at 30°C, single colonies were picked and the sequence of their *aroQ*_δ_ genes was determined. From the eight combinatorial libraries, a total of 111 members growing on selective plates were sequenced. Fig. 5B summarizes the pattern of residues found in MtCM variants that enable survival under stringent selection conditions in the presence of MtDS.

**Figure 6:**
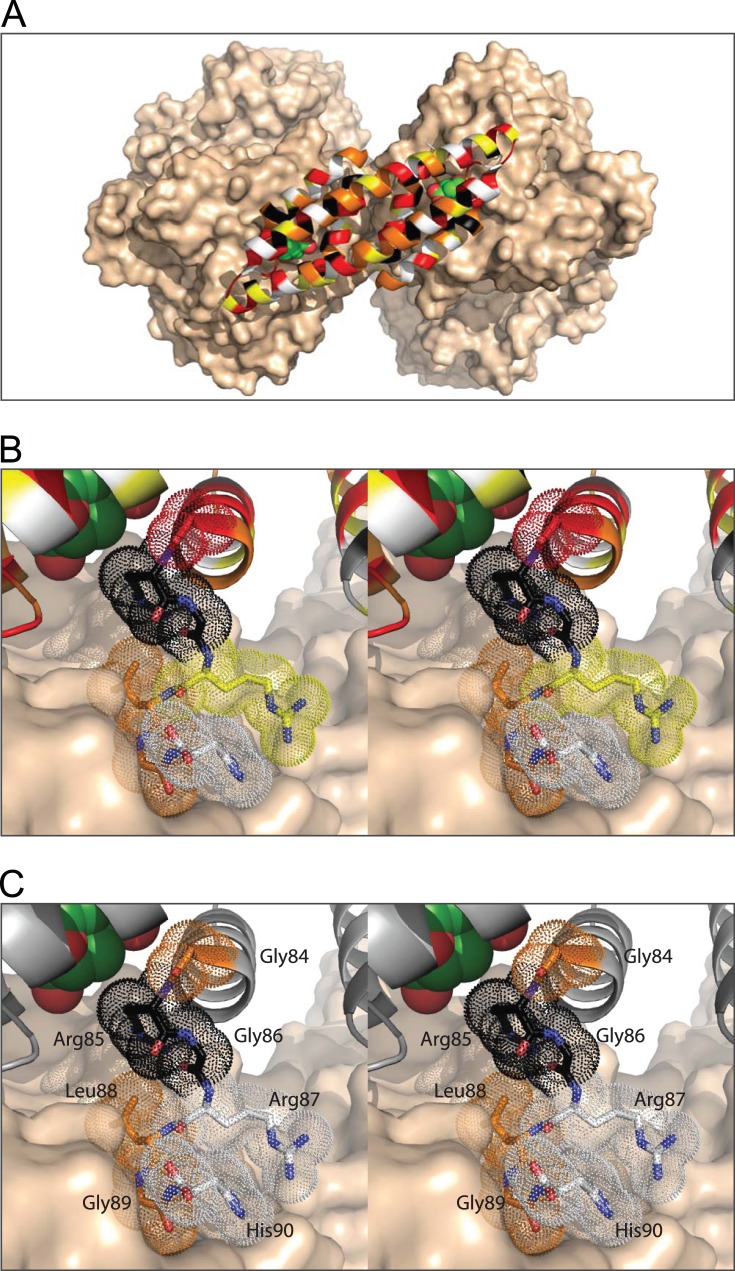
Comparison of phylogenetic and experimental amino acid conservation patterns at the interface between MtCM and MtDS. Conservation patterns in MtCM are illustrated with the crystal structure of the MtCM-MtDS complex (PDB: 2W1A). The color code for individual residues indicates the level of sequence conservation (black, 100%; red, ≥75%; orange, ≥50%; yellow, ≥33%; white, <33% identity). (A) Overview with MtCM positions colored as in the phylogenetic alignment in Fig. 3B. MtDS is shown in wheat surface representation, MtCM in cartoon mode. Fully conserved residues (black) are generally clustered around the active site with bound **7** (shown as space-filling model with green carbons), whereas the solvent-exposed residues typically show low conservation (white and yellow). (B, C) Wall-eyed stereogram of a close-up of the contact area between the C-terminal region of MtCM and MtDS, with ligand **7** depicted in the top left corner as in (A). The C-terminal loop hooks onto the MtDS surface. In (B), the phylogenetic color coding as in (A) is used for the segment from positions 84–90, which is highlighted with dotted spheres for the individual side chains. In (C), the color coding for the same segment represents the conservation pattern found by experiment in selected MtCM variants in the presence of MtDS.

All selected clones retained a small amino acid at position 84 (Gly, Ala, Thr, Ser). This coincides with the conservation of a small residue (Gly, Ser, Cys) in the alignment of natural AroQ_δ_ sequences (Fig. 3B). The crystal structure of the MtCM-MtDS complex (Fig. 6) provides a rationale for this finding. Gly84 is buried in the protein, leaving little space for larger amino acids at this position. The Arg85-Gly86 dyad was fully conserved in all experimentally selected clones (Fig. 5B), as well as in natural AroQ_δ_ proteins (Fig. 3B), and thus might be crucial for complex formation. Alternatively, these residues may play a role in the catalytic machinery of MtCM, which was probed in a separate experiment described below. Arg85 makes hydrophobic contacts to the transition state analog **7** *via* its apolar methylene groups and also to the MtDS surface (Fig. 6) [10]. It is completely engulfed by surrounding residues and by **7**, except for the charged head group, which is partially solvent accessible. Inspection of the crystal structure of the complex also provides a rationale for the total conservation of Gly86 found in the experiment. Even though the α-carbon of Gly86 is surface exposed and enough space would potentially be available to accommodate larger residues, Gly86 is probably retained because it is the only residue that can prevent unfavorable interactions of other C-terminal amino acids with the MtCM catalytic core. This conclusion is supported by a Ramachandran analysis where the φ/ψ-angles of Gly86 place this residue far away from the allowed regions for all other amino acids. Thus, Gly86 appears to be an ideal choice because it can break the last helix, allowing the C-terminal residues to bend away from the protein core and thereby making them available for interactions with MtDS (Fig. 6) [10].

Residue Arg87 is only moderately conserved phylogenetically (Fig. 3B) and also exhibits high variability in our selection experiments, including substitutions by smaller and other polar residues (Fig. 5B). Space constraints may disfavor amino acids bulkier than arginine, since clearly less Phe, Trp, or Tyr were selected. In the crystal structure of the MtCM-MtDS complex, Arg87 makes only few contacts to other residues in MtCM and none to MtDS (Fig. 6), suggesting that it is less important for activity enhancement by MtDS. In contrast, Leu88 is almost completely buried by other residues from MtCM and MtDS (Fig. 6). In this context, it is remarkable that several other amino acids are tolerated at this position. Besides the preferred Leu, other apolar amino acids like Ala, Pro, Val, Ile, and Met were found, suggesting that the size of the residue is less important at this site of the interaction interface than the preservation of hydrophobic contacts.

Similar to Leu88, Gly89 makes close contacts to residues of both MtCM and MtDS (Fig. 6). From the selected amino acid pattern at this position, small residues seem preferred for high CM activity of the complex. Interestingly, besides mostly Gly, Ala, and Ser, many of the selected variants terminated at position 89 (Fig. 5B). Thus, larger residues appear to be more detrimental to complex formation than the absence of the last two C-terminal amino acids. About one third of the natural AroQ_δ_ proteins in the multiple sequence alignment (Fig. 3B) also terminate at this position, corroborating our observations. Even though His90 interacts with four MtDS residues in the crystal structure (Glu396, Arg399, Arg461, and Asp462; PDB: 2W1A), it is not conserved among the selected mutants. The properties of the side chains that act as functional substitutes range from very small to very large and from hydrophobic to charged (Fig. 5B).

Overall, the pattern of residues emerging from the selection experiments in the presence of MtDS mirrors the conservation pattern in the multiple sequence alignment of AroQ_δ_ proteins (Fig. 3B; visualized in Fig. 6). In general, conservation of MtCM residues could either mean that they are important for the AroQ_δ_-specific activation by interaction with MtDS or that they are required for the intrinsic catalytic machinery of MtCM. To distinguish between these two possibilities the seven C-terminal positions of MtCM were probed in an independent experiment for their direct involvement in CM catalysis. This was accomplished by surveying the complementation ability of the randomized MtCM variants under less stringent conditions, where formation of a complex with MtDS is not required for survival and growth on minimal plates. Specifically, plating onto the only mildly selective agar plates M9c +F +500 ng/mL Tet allows for good growth of clones with wild-type MtCM in the host KA12/pKIMP-UAUC, even in the absence of MtDS (Fig. 4).

Five representative previously constructed libraries (CT7, GRGR, LGH, GRG, and RLGH; Fig. 3C) were transformed into KA12/pKIMP-UAUC and between 0.16% and >50% of the library members were able to form colonies on M9c +F +500 ng/mL Tet. Sequencing of 106 complementing clones yielded the conservation pattern shown in Fig. 5C. From the high frequency of small residues at position 84, and the almost 100% conservation found for Arg85 and Gly86, we conclude, that residues 84 to 86 are not specifically responsible for the AroQ_δ_-typical activation through complex formation. Instead, these residues are required for the basic catalytic machinery in MtCM or for the integrity of its structure. Such a role is conceivable, as Arg85 contacts the ligand directly and Gly86 allows for kinking the polypeptide chain at the C-terminus to maintain an unobstructed active site.

The positions C-terminal to Gly86 do not seem to be essential for MtCM activity, since they show a rather random distribution of amino acids. A plot relating the frequencies of conserved residues selected in complexed *vs.* free MtCM (Fig. 5D) reveals that the complexed MtCM shows a preference for Met at position 88 followed by Leu. Furthermore, a Gly (or a stop codon) is strongly favored at position 89 for the complex, whereas free MtCM shows a fully random distribution of residues here. Overall, the intrinsic low CM activity of free MtCM is more tolerant to C-terminal mutations, as apparent from the many columns with small negative values in Fig. 5D. In contrast, the conservation of residues specifically in the presence of MtDS pinpoints critical hinges and contact areas involved in productive transmission of conformational changes from the interface of the enzyme complex to the active site, as already discussed in the previous section in the context of the MtCM-MtDS structure.

### Properties of C-terminally randomized MtCM variants

**Figure 7:**
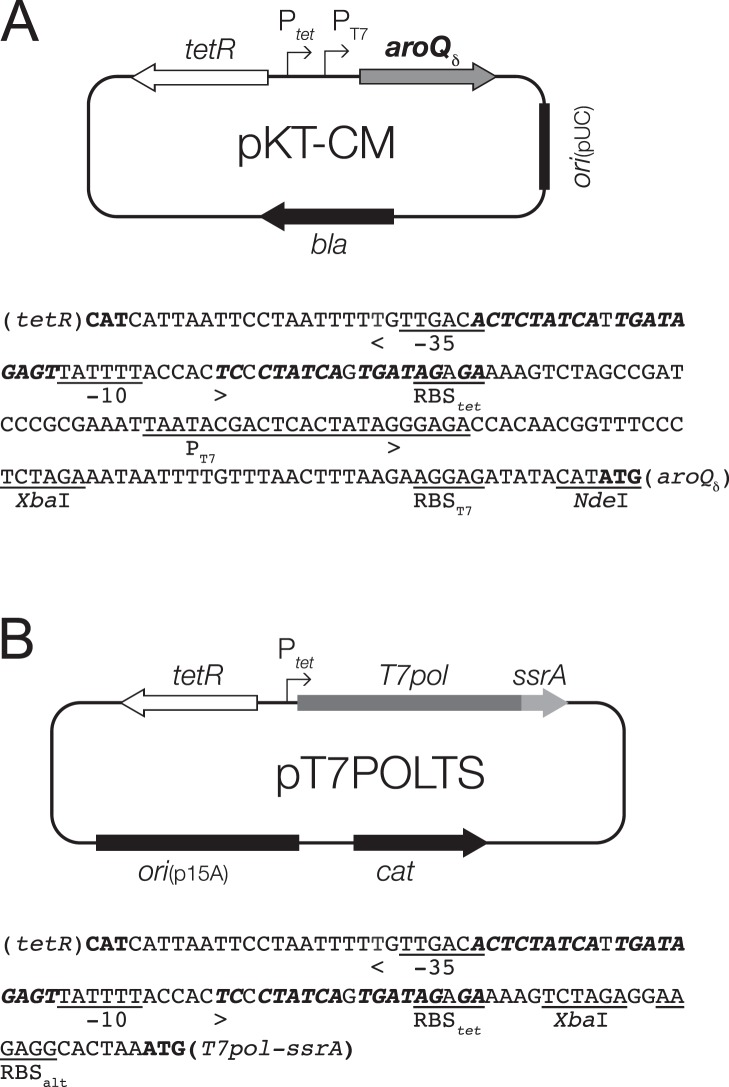
Overview of the plasmid-based T7 RNA polymerase gene expression system. (A) Map and relevant promoter sequence of library plasmid pKT-CM. This plasmid was used for both *in* *vivo* selection (relying on P*_tet_*, see also Fig. 4B) and *in* *vitro* overproduction of the MtCM variants (using P_T7_). The sequence of the P*_tet_*P_T7_ tandem promoter is given with the binding sites for the Tet-responsive TetR repressor highlighted in bold italics and the start codons of the reading frames in bold roman type [49]; underlined are relevant restriction sites, the ribosomal binding site (RBS*_tet_*), −35 and −10 regions of P*_tet_* [49] and the RBS_T7_ and promoter P_T7_ from phage T7 [23], [50]; start and direction of transcription is marked by an arrowhead [22]. (B) Map and relevant promoter sequence of the T7 RNA polymerase plasmid pT7POLTS. The plasmid carries the p15A origin of replication derived from pACYC184 [51], *cat* encoding chloramphenicol acetyltransferase, and a P*_tet_* controlled T7 RNA polymerase gene (*T7pol*) translationally fused at its 3′ end to the sequence for the SsrA degradation tag. In the absence of Tet, TetR binding to its operator sites (highlighted as in panel A) blocks gene expression from P*_tet_* and any T7 RNA polymerase produced due to low-level leaky transcription is effectively eliminated by SsrA-mediated Clp proteolysis, thereby suppressing basal polymerase activity. Provision of Tet releases TetR from the operator, resulting in intracellular polymerase levels higher than can be degraded efficiently by the Clp proteases [41]. For efficient translation, the alternative RBS_alt_ can be used. The accumulating polymerase then directs massive transcription from P_T7_ controlled genes, such as *aroQ_δ_* on pKT-CM. The entire nucleotide sequence of pT7POLTS is provided as S2 Fig.

To examine the impact of C-terminal amino acid exchanges on the kinetic properties of MtCM, several selected enzyme variants were overproduced and purified. The library plasmid pKT-CM features, in addition to P*_tet_* required for gene expression during *in* *vivo* selection, also the much stronger T7 promoter in tandem configuration (Fig. 7A). Typically, this promoter is used for high-level gene expression in conjunction with an engineered *E. coli* strain possessing a chromosomally integrated gene for T7 RNA polymerase controlled by the *lac*-promoter [23]–[25]. To exclude *a priori* any contamination of the purified proteins by CM activity from the two endogenous *E. coli* enzymes, we developed a new, generally applicable gene expression strategy that relies on a plasmid-borne T7 RNA polymerase gene, allowing for convenient overproduction of the MtCM variants in our CM-deficient mutant strain KA12 (Fig. 7B).

The new plasmid pT7POLTS (*ori*_p15A_) is compatible with pKT-CM (*ori*_pUC_), carries a chloramphenicol resistance marker and the tetracycline repressor, which controls expression of an adjacent P*_tet_* regulated T7 RNA polymerase gene in response to the concentration of the inducer Tet. A problem often encountered with T7 RNA polymerase-driven gene expression is an undesirable high basal activity (“leakiness”) prior to addition of inducer [25]. This is typically caused by trace amounts of lactose that contaminate complex media components to varying degrees, resulting in low-level induction of the chromosomal *lac* promoter-controlled T7 RNA polymerase gene [26]. In our system, any background expression is efficiently reduced with an in-frame translational fusion of the C-terminus of the polymerase to an SsrA peptide tag that directs the T7 RNA polymerase mostly to the ClpXP protease system [27], [28]. In fact, even though the modified polymerase gene was placed on the intermediate high copy-number plasmid pT7POLTS, there was no apparent gene expression in the absence of the inducer [29]. Upon induction with 2 µg/mL Tet, T7 RNA polymerase production –now presumably exceeding the degradation capacity of ClpXP– leads to strong expression of the target gene on the library plasmid pKT-CM from its tandem P*_tet_*P_T7_ promoter system (Fig. 7).

Table 1 lists MtCM library variants competent in MtDS activation that were chosen for further characterization. After production and purification by metal affinity chromatography, the electrophoretically homogeneous proteins were assessed for their structural integrity by circular dichroism (CD) spectroscopy. All clones showed spectra comparable to wild-type MtCM, with a dominant α-helical structure as apparent from the typical relative minima at 208 and 222 nm. Also, the expected molecular masses of the variants were confirmed by electrospray ionization mass spectrometry within the error of the experiment (±5 Da).

**Table 1:** Chorismate mutase activity of wild-type MtCM (wt) and selected MtCM library variants.^a.^

Variant	C-Terminal positions 84–90^b^	*k*_cat_/*K*_m_ (M^-1^ s^-1^)^c^	Fold activation by MtDS^d^	Remaining activity of complex +Phe +Tyr (%)^e^
wt	GRGRLGH	790±120	131±43	7.8±2.3
1–6	GRGPL*	1350±200	4.5±1.0	45±10
2–8	GRGHL*	1050±160	4.8±1.0	18±5
3–16	GRGHLGH	1150±170	82±16	3.7±0.8
4–4	GRGILGH	640±100	24±4	3.6±1.7
4–5	GRGKLGH	630±100	106±16	4.1±0.9
2–4	GRGKLGT	620±90	40±7	7.0±1.2
ST–46	GRGRLTR	870±130	7.9±2.0	14±6
2–17	GRGQLGC	1150±170	6.7±1.1	22±3

a Details to the assay conditions and calculation of individual parameters and their standard deviations are provided in Materials and Methods.

b An asterisk indicates premature termination (the last residue is Leu88 for variants 1–6 and 2–8).

c Catalytic efficiency of MtCM variant alone (in the absence of MtDS).

d The apparent activation factor was estimated as described previously [10], as the ratio of CM initial velocities of the MtCM-MtDS complex (*v*_0 (MtDS+MtCM)_), normalized by MtCM-variant and chorismate concentrations, over *k*_cat_/*K*_m_ for free MtCM.

e Ratio of initial velocity *v*_0 (MtDS+MtCM)_ as in footnote ^c^, but measured in the presence of 100 µM Phe and 100 µM Tyr, divided by *v*_0 (MtDS+MtCM)_ obtained in the absence of these feedback inhibitors.

In the absence of MtDS, all variants exhibited a catalytic efficiency (*k*_cat_/*K*_m_) within a factor of 2 of the wild-type MtCM (Table 1). They all are activated *in* *vitro* by MtDS by more than a factor of 4, which apparently suffices for complementation under the stringent *in* *vivo* selection regime. The magnitude of activation correlates with some sequence features found in the variants. It appears that premature termination at position 88 reduces the activation by MtDS by about 30-fold (for variants 1–6 and 2–8). In contrast, retaining a positively charged amino acid in place of Arg87 (*i.e.*, His or Lys in clones 3–16 or 4–5, respectively) correlates with high activation factors, suggestive of a functionally preferred electrostatic interaction in the complex *in* *vitro*, whereas a hydrophobic (Ile, variant 4–4) or uncharged polar residue (Gln, variant 2–17) at this position weakens MtDS activation. The regulatory behavior of the purified MtCM variants was also assessed in response to the presence of the metabolic end products of the chorismate mutase branch. Interestingly, a strong response to inhibition by Phe and Tyr generally coincides with a high degree of activation by MtDS (Table 1).

**Figure 8:**
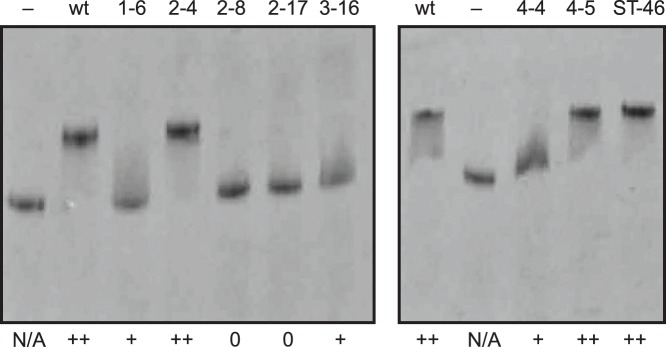
Ability of MtCM variants to shift the MtDS band during native PAGE. The ability of the variants, identified above the lanes, to shift the MtDS band was scored as++(full shift),+(weak shift), and 0 (no shift). As controls, samples without MtCM (–) and with wild-type MtCM (wt) were included. The samples were applied to a 12.5% native polyacrylamide gel at equimolar concentrations (4 µM of each MtCM and MtDS in the loading mixture). The pH of the gel was 8.8; the pI of MtDS is 6.13. MtCM variants do not appear as discrete bands on these gels, since their pI lies above the pH of the electrophoresis buffer. The pI was calculated based on the sequence using the Wisconsin program package from Accelrys (San Diego, USA). N/A, not applicable.

To survey the physical interaction between MtDS and the different MtCM variants band-shift experiments with native polyacrylamide gel electrophoresis (PAGE) were performed (Fig. 8). Although MtCM variants do not appear as discrete bands because their pI is above the pH of the gel, we observed a full shift of the MtDS band if wild-type MtCM is present. Like the wild-type control, the highly activated variants 4–5 and 2–4 cause maximum MtDS shifts, but also the poorly activated variant ST-46. Thus, even though physical interaction is necessary, it is not sufficient for high catalytic activatability by MtDS. The extra positive charge (Arg90) at the C-terminus of ST-46 might be responsible for the tight, but functionally modest interaction with MtDS. In fact, an Arg90 modeled into the crystal structure of the MtCM-MtDS complex [10] could form a salt bridge with the C-terminal Asp462 of MtDS, either to its side chain or its free C-terminal carboxylate. Even though all variants are catalytically activated (to some extent) *in* *vitro* and *in* *vivo* by MtDS, no physical interaction with the partner enzyme was apparent for MtCM versions 2–8 and 2–17 under the prevailing conditions of the native PAGE, suggesting that these variants bind more weakly to MtDS.

**Figure 9:**
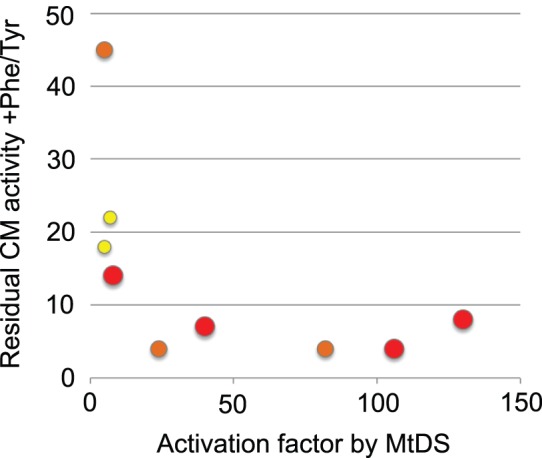
Correlation between MtDS interactions and sensitivity to feedback inhibition of MtCM variants. The plot shows a graphical correlation of the salient features of the selected MtCM library variants (and the wild type) from Table 1 and Fig. 8. All variants that are activated by MtDS by more than 20 fold also show high sensitivity of the complex to feedback inhibition by Phe and Tyr (*i.e.* show low residual activity +Phe/Tyr). Conversely, lower sensitivity to feedback inhibition correlates with poor activation by MtDS and with a lower potential to shift the MtDS-band on native PAGE. Circle size and color represent a full (large, red), partial (intermediate, orange), or no shift of the MtDS band (small, yellow).

In summary, the selection experiments with C-terminally randomized MtCM libraries resulted in a set of distinct regulatory variants with roughly similar intrinsic basal CM activities but varying widely in their potential to become activated by MtDS. Simultaneously, the activation potential correlates roughly with the degree of the physical interaction with MtDS observed by native PAGE and the sensitivity to feedback inhibition of the complex, as illustrated in Fig. 9.

## Conclusions

We have applied the strategies of directed evolution –*i.e.*, randomizing mutagenesis, selection *in* *vivo*, and analysis of surviving library members– to quickly survey essential protein-protein interactions in the MtCM-MtDS enzyme complex. The pattern of sequence conservation for MtCM’s seven C-terminal residues that we observed in our laboratory evolution experiments essentially coincides with the pattern that emerged during natural evolution of homologous CMs. From two complementary selection experiments, carried out in the absence and in the presence of MtDS, we were able to discriminate between residues needed mainly for the basic integrity of AroQ_δ_ CMs and those required for catalytically productive complex formation, respectively.

Because the biosynthesis of the aromatic amino acids Phe and Tyr is energetically very costly for organisms [30] it needs to be tightly regulated, particularly at metabolic branch point reactions and at irreversible steps, such as the one catalyzed by CM [31]. It was hypothesized that the level of CM activity in an *M. tuberculosis* cell is regulated by rapid dynamic control of the ratio between the poorly active free MtCM and the over 100 fold more active MtCM when in complex with MtDS [10]. Specifically, if intracellular Phe and Tyr is abundant, these shikimate pathway end products could influence this equilibrium by binding to allosteric effector sites in the MtCM-MtDS assembly, resulting in a further increase of the already high (140 nM) apparent dissociation constant *K*_d, app_ of the hetero-octameric complex [10]. The examination of the MtCM variants and their interactions with MtDS described here support a regulatory mechanism that involves shifting the equilibrium between free and complexed MtCM. In general, a poor ability of an MtCM variant to bind to MtDS on a native gel correlates with both a rather modest MtDS-mediated CM activity increase and a poor response to feedback inhibition.

Finally, high-resolution structures of some of the strongly deregulated variants selected here, such as clones 1–6 or 2–17, could inform the rational design of molecules that interfere with protein-protein interactions [32]–[35]. Drugs that target *Actinomycetales*-specific CM-DS complexes [10], [15] may cause an activity decrease by blocking the protein-protein interface through direct competitive binding, or by stabilizing a non-productive conformation of one partner protein, as may be assumed by some of the MtCM variants found in this work. In principle, our evolutionary strategy is applicable for characterizing druggable regions in any protein complex for which a selectable function depends on protein-protein interactions.

## Materials and Methods

### Strains, Plasmids, and General DNA Procedures

*E. coli* strain KA12 [21], [36] was used for *in* *vivo* assays, protein production and standard cloning. Details on plasmids pKTCM-HC, pKTDS-H, and pKTDS-HN [10], pKTH-400-5 [17], [29], pKIMP-UAUC [21], and pKSS [37] were published previously. Jetquick spin columns from Genomed (Brunschwig AG) and NucleoSpin cups from Macheréy-Nagel were used for DNA purification. Other DNA manipulations were performed using standard procedures [38]. Restriction endonucleases, T4 DNA ligase and calf intestinal phosphatase were from New England Biolabs. Oligonucleotides were synthesized by Microsynth. DNA sequencing using the BigDye terminator cycle sequencing kit was performed on an ABI PRISM 3100-Avant Genetic Analyzer (Applied Biosystems), unless stated otherwise. All other chemicals were from Fluka/Sigma-Aldrich.

### Reengineering of the Chorismate Mutase Selection System

Tight control of MtCM gene expression *in* *vivo* was achieved by reengineering the selection system towards a tetracycline-inducible promoter system. pKTCM-HCDXhoI was constructed from pKTCM-HC [10] by PCR amplification of a 270 bp fragment using oligonucleotides 293-DXhoI-S (5′-TGGAAATCGTGGAGTCCCAACCTGT) and 204-MTCM2N (5′-CGATAACTCGAGGTGACCGAGGCGGCCACGGCCCAAT, employed restriction site underlined). The obtained PCR fragment was subsequently used as a mega-primer together with oligonucleotide 206-MTCM2S (5′-ACCGATGTCATATGCGTCCAGAACCCCCACATCA) on pKTCM-HC as a template. The *Nde*I/*Xho*I-digested fragment (318 bp) was ligated to the 4561 bp *Nde*I-*Xho*I fragment from pKTCM-HC, yielding the 4879 bp pKTCM-HCDXhoI plasmid. This plasmid was used to construct pMG242, containing a shortened but functional version of the *aroQ*_δ_ gene, which encodes an MtCM variant starting at Met5. Ligation of the *Nde*I/*Xho*I-digested product (261 bp) of the PCR with oligonucleotides 297-SHO-S (5′-CAACATATGCTGGAGTCCCAACCT) and 204-MTCM2N on template pKTCM-HCDXhoI to the 4561 bp fragment of the *Nde*I/*Xho*I-digested pKTCM-HC plasmid yielded the 4822 bp pMG242. Plasmid pKSS-TM4 was used as the template for library construction. pKSS-TM4 contains a non-functional portion of the *aroQ*_δ_ gene on a pKSS backbone [37], lacks the restriction sites used later for library cloning, and was assembled from a PCR fragment generated using oligonucleotides 341-MCMN-SacI-S (5′-CTCACGAGCTCACCATCATCATCACCACTTCTTCTGGTATGCTCGAGTCCCAACCTGT) and 342-MCM-KpnI-N (5′-TACTTGGTACCTTAGCGGCCCAAGCGCAAAAGCAGGATGGCCAGA) on template pMG242. The 278 bp *Kpn*I-*Sac*I fragment was ligated into the correspondingly cut pKSS fragment of 2855 bp, yielding pKSS-TM4 (3133 bp). Plasmid pKTCMtet2-HC (3058 bp) contains the *aroQ*_δ_ gene driven by a tandem P*_tet_*P_T7_ promoter system, as well as the *tetR* gene for controlled repression of the *tet* operator within P*_tet_*. It was obtained by ligating the 261 bp *Nde*I-*Xho*I fragment of pMG242 to the 2797 bp *Nde*I-*Xho*I fragment of plasmid pKTH-400-5 (3100 bp) [17], [29]. To generate an *aroQ*_δ_-negative control and as acceptor vector for the libraries, plasmid pKTCTET-0 (4096 bp) was constructed by ligating the 2797 bp *Nde*I-*Xho*I fragment of pKTCMtet2-HC with the 1299 bp *Nde*I-*Xho*I stuffer fragment from pMG242-0. Plasmid pMG242-0 (5860 bp) is derived from pMG242 by inserting a Tet resistance stuffer fragment. It was constructed in two steps, first by introducing a silent *Asc*I site into the MtCM gene of pMG242, yielding pMG242-sil, and subsequently, by cloning an *Asc*I/*Xho*I-digested Tet resistance determinant fragment into the *Asc*I/*Xho*I-digested pMG242-sil. For the construction of pMG242-sil, a 193 bp fragment generated with primers 299-Ascsil-S (5′-TTAGTCAAGCGGCGCGCCGAGGTTTCCAAGGCCAT-3′) and 204-MTCM2N on template pMG242 was used as a megaprimer for a second PCR on the same template together with 297-SHO-S, giving a 278 bp fragment. This fragment was cut with *Nde*I and *Xho*I (261 bp) and inserted into the *Nde*I/*Xho*I-digested pKTCM-HC vector backbone (4561 bp) to give pMG242-sil (4822 bp). The fragment carrying the Tet resistance determinant was amplified from a DNA sequence of pBR322 [39] with primers 302-STUFF (5′-CAAACTCGAGCCGTGTATGAAATCTAA-3′) and 162-STS (5′-CAAAGGCGCGCCCATTCAGGTCGAGGT-3′). The resulting 1222 bp PCR fragment was digested with *Xho*I and *Asc*I to yield a 1207 bp stuffer fragment (encoding the Tet resistance determinant) that was then ligated with the 4653 bp *Xho*I/*Asc*I-digested pMG242-sil to give plasmid pMG242-0 (5860 bp). Plasmid pKTNTET was constructed by restriction digestion of plasmids pMG244 and pKTCTET-0 with *Nde*I and *Spe*I. The respective 296 bp and 2765 bp fragments were ligated and yielded plasmid pKTNTET (3061 bp). pKTNTET encodes the His_6_-tagged MtCM sequence defined in this work as wild-type (wt) MtCM (*i.e.*, MtCM carrying an N-terminal Met-His_6_-Ser-Ser-Gly sequence fused to Met5 of entry Mtu in Fig. 3C) but otherwise has the same structure as the library plasmids “pKT-CM” and was therefore used as the positive control in *in* *vivo* complementation tests; its entire nucleotide sequence is provided as S1 Fig. pMG244 was constructed from ligation of the 4529 bp *Nde*I-*Spe*I-fragment of pMG243 to the 296 bp *Nde*I/*Spe*I-digested PCR fragment obtained from amplification using oligonucleotides 201-MTCM2HS (5′-ACCGATGTCATATGCACCATCATCATCATCATTCTTCTGGTATGCTCGAGTCCCAACCT) and 203-MTCM2CN (5′-CGATACACTAGTTATTAGTGACCGAGGCGGCCACGGCCCAAT) on template pKTCM-HC. pMG243 (4879 bp) was constructed from the 4561 bp *Nde*I/*Xho*I fragment of pKTCM-HC and a 318 bp *Nde*I/*Xho*I-digested PCR fragment obtained from amplification of pKTCM-HCDXhoI using oligonucleotides 294-RTOK (5′-GCAAGGCCAAAATGGCGTCCGGT) and 204-MTCM2N, where the 155 bp PCR product was used as primer together with oligonucleotide 206-MTCM2S, also on template pKTCM-HCDXhoI (crude PCR product length, 338 bp; *Nde*I/*Xho*I-digested fragment, 318 bp).

To carry out *in* *vivo* selection experiments for MtCM variants able to interact with MtDS, the gene for the DAHP synthase was provided on the separate, compatible plasmid called pKIMP-ACG. pKIMP-ACG is based on pKIMP-UAUC [21] and carries the tandem P*_sal_*P_T7_ controlled *aroG* from *M. tuberculosis* encoding MtDS, in addition to the helper functions tyrA* and *pheC*, and also a copy of *nahR* encoding the transcriptional activator of the *sal* promoter [40]. For its construction, plasmid pKIMP-UAUC was digested with *Spe*I and subsequently treated with calf intestinal phosphatase. The product was completely digested with *Nar*I. pKTDS-H [10] was cut to completion with *Spe*I and partially digested with *Sph*I. Since the resulting desired 2592 bp and undesired 2515 bp fragments were not separable by agarose gel electrophoresis, they were used as a mixture for subsequent ligation (in equimolar ratio) with both the 4993 bp *Spe*I-*Nar*I fragment of pKIMP-UAUC and the self-annealing oligonucleotide pair (334-trpAfw 5′-CAGCTTAGCCCGCCTAATGAGCGGGCTTTTTTTGG and 335-trpArv 5′-CGCCAAAAAAAGCCCGCTCATTAGGCGGGCTAAGCTGCATG), which forms a *trpA* transcriptional terminator (with *Nar*I and *Sph*I-compatible ends) and which was included to circumvent transcriptional coupling of the *pheC* and *nahR* genes. The ligation product was used to transform competent KA12 cells and a clone (pKIMP-ACG; 7623 bp) containing the correct (2592 bp) fragment from pKTDS-H was identified by sequencing.

### Construction of MtCM Libraries

The eight partially randomized gene libraries CT3, CT4, CT7, GRG, GRGR, LGH, CT-L, and RLGH were constructed by PCR amplification using pKSS-TM4 as a template and the sense primer 352-CTLib-S (5′- CCTTGTTCATATGCACCATCATCATCACCACTCTT) combined with either 351-CT3-N (5′-TCGACTACTAGTTATTAGTGACCGAGNNNNNNNNNACCAAGACGCAAAAGCAGGATGGCCAGAT for CT3), 428-CT-GRGrv (5′-GGTTAAAGCTTCCGCAGCCACTAGTTATTAGTGACCGAGTCGNNNNNNNNNAAGACGCAAAAGCAGGATGGCCAGAT for GRG), 350-CT4-N (5′-TCGACTACTAGTTATTANNNNNNCAGNNNGCCNNNACCAAGACGCAAAAGCAGGATGGCCAGAT for CT4), 349-CT7-N (5′-TCGACTACTAGTTATTANNNNNNNNNNNNNNNNNNNNNAAGACGCAAAAGCAGGATGGCCAGAT for CT7), 367-CT-LGHrv (5′-TCGACTACTAGTTATTANNNNNNNNNTCGACCACGACCAAGACGCAAAAGCAGGATGGCCAGAT for LGH), 365-CT-Lrv (5′-TCGACTACTAGTTATTAGTGACCNNNTCGACCACGACCAAGACGCAAAAGCAGGATGGCCAGAT for CT-L), 477-CT-GRGRrv (5′-GGTTAAAGCTTCCGCAGCCACTAGTTATTAGTGACCGAGNNNNNNNNNNNNAAGACGCAAAAGCAGGATGGCCAGAT for GRGR), or 424-CT-RLGHrv (5′-GGTTAAAGCTTCCGCAGCCACTAGTTATTANNNNNNNNNNNNACCACGACCAAGACGCAAAAGCAGGATGGCCAGAT for RLGH). The 315 bp (libraries CT3, CT4, LGH, CT7, and CT-L) or 328 bp (for libraries GRG, GRGR, and RLGH) fragments were restriction digested with *Nde*I and *Spe*I and the resulting 296 bp library fragment (for N-terminally His_6_-tagged MtCM variants) was ligated in equimolar concentration (typically 0.2 pmol) to the 2765 bp fragment of the correspondingly cut pKTCTET-0 acceptor vector. The ligated library plasmids, denoted as “pKT-CM”, were desalted and transformed into electrocompetent KA12/pKIMP-ACG or KA12/pKIMP-UAUC cells.

### *In Vivo* Selection for MtCM Variants

The transformed cell suspension (with either KA12/pKIMP-ACG or KA12/pKIMP-UAUC transformants) was washed three times with 1×M9 salts (6 mg/mL Na_2_HPO_4_, 3 mg/mL KH_2_PO_4_, 1 mg/mL NH_4_Cl, and 0.5 mg/mL NaCl, pH 7) [38] and spread out on M9c minimal medium agar plates (consisting of 1×M9 salts, additionally containing 0.2% (w/v) d-(+)-glucose, 1 mM MgSO_4_, 0.1 mM CaCl_2_, 5 µg/mL thiamine-HCl, 5 µg/mL 4-hydroxybenzoic acid, 5 µg/mL 4-aminobenzoic acid, 1.6 µg/mL 2,3-dihydroxybenzoic acid, 20 µg/mL l-Trp, 100 µg/mL sodium ampicillin, 20 µg/mL chloramphenicol, and 1.5% agar) or M9c +F +Tet^500 ng/mL^ (in addition to M9c, this medium also contains 20 µg/mL l-Phe and 500 ng/mL Tet) for selection. For determination of the library size, cells were also plated onto supplemented minimal medium M9c +FY plates, which in addition to the M9c medium ingredients also contain 20 µg/mL of each l-Tyr and l-Phe. Analysis of plates occurred after two (M9c +FY) or three days (M9c or M9c +F +Tet^500 ng/mL^) incubation at 30°C. Single colonies grown on selective plates were purified on LB +Amp +Cam plates (150 µg/mL Na-ampicillin; 30 µg/mL chloramphenicol). Isolated pKT-CM plasmid DNA was sequenced using oligonucleotide 131-TERM (5′-CCCTCAAGACCCGTTTAGA), except for KA12/pKIMP-UAUC libraries, where plasmid isolation and sequencing using oligonucleotide T7 (5′- TAATACGACTCACTATAGG) was performed by Microsynth. The complementation ability of a number of pKT-CM plasmids selected in the presence of MtDS was confirmed by retransformation into CaCl_2_-competent KA12/pKIMP-ACG cells and reassessing their growth phenotypes at 30°C on M9c, M9c +Tet^80 ng/mL^, M9c +F (20 µg/mL l-Phe) and M9c +FY minimal plates. pKTNTET in KA12/pKIMP-ACG was used as positive control; pKTNTET in KA12/pKIMP-UAUC and pKTCTET-0 in KA12/pKIMP-ACG served as partially and fully negative controls, respectively.

### A Generally Applicable Plasmid-borne T7 RNA Polymerase System

Plasmid pT7POLTS (5901 bp) was used as a new and convenient plasmid-based T7 RNA polymerase gene expression system. It contains a P*_tet_* controlled T7 RNA polymerase gene genetically fused in-frame to the DNA encoding a C-terminal SsrA degradation tag. The SsrA tag targets the polymerase to cellular degradation machineries, such as the ClpXP or ClpAP protease complexes [27], [28]. Thereby, intracellular T7 RNA polymerase concentrations are kept very low in the uninduced state, preventing circumstantial toxicity during the growth phase of the production culture by the gene to be overexpressed. After induction with Tet, SsrA-tagged T7 RNA polymerase accumulates, presumably because its level exceeds the degradation capacity of the protease systems [41], leading to massive transcription from the P_T7_ controlled gene of interest that is present on a second plasmid.

In *E. coli* strains, which do not carry an endogenous Tet resistance, gene expression from P*_tet_* was shown to be homogeneous in each cell, and it responded over 2 to 3 orders of magnitude with little cooperativity (in the case of the moderate-copy number pT7POLTS) to increasing Tet concentrations up to 100 ng/mL, before the antibiotic became toxic [22]. For Tet resistant *E. coli* strains, such as strain KA12 used in this work harboring the *tetR-tetA* regulatory system from Tn*10*, the dose-response profile is fully linear and shifted to higher Tet concentrations up to 5 µg/mL [22]. The latter is due to the fact that the TetA resistance determinant is an antiporter that lowers the intracellular inducer concentration by coupling Tet efflux to the H^+^ gradient across the membrane.

Additionally, pT7POLTS carries a p15A origin of replication, a chloramphenicol resistance gene, and the *tetR* repressor gene responsible for tight control of T7 RNA polymerase transcription. The assembly of pT7POLTS (previously also referred to as pAC-Ptet-T7pol-S) is described in detail elsewhere [29]; its entire nucleotide sequence is listed as S2 Fig. We expect our pT7POLTS system, which circumvents the need for a chromosomally integrated T7 RNA polymerase gene, to be of general use for T7-promoted gene expression also beyond this project.

### Gene Expression and Protein Purification

Production of N-terminally His_6_-tagged MtCM variants (Met-His_6_-Ser-Ser-Gly tag fused to Met5 of the Mtu sequence in Fig. 3C) was carried out using KA12/pT7POLTS cells transformed with pKTNTET (for wild type) or pKT-CM plasmids (for library members). Cultures were grown in 500 mL LB medium containing 150 µg/mL Na-ampicillin and 30 µg/mL chloramphenicol at 30°C and gene expression was induced with 2 µg/mL Tet at an OD_600_ of 0.3–0.5. The crude lysate was obtained following published protocols [42], except for omitting the RNase A and DNase I treatment. The crude lysate was provided with imidazole (10 mM) and loaded onto a column packed with an equilibrated His-Select Nickel Affinity Gel (Sigma). The AroQ_δ_ variant was eluted with 250 mM imidazole and dialyzed against 20 mM potassium phosphate, pH 7.5.

MtDS protein needed for kinetic assays and native gels was produced in the His-tagged format (containing a Met-His_6_-Ser-Ser-Gly sequence appended to the natural N-terminus) from KA13/pKTDS-HN as described earlier [10], [43].

The concentration of the purified enzymes was determined by the Micro BCA Protein Assay Reagent Kit (ThermoFisher, formerly Pierce) using BSA as a standard or a calibrated Bradford assay with BSA values corrected for MtCM and MtDS-specific absorption [10].

### Structural Investigation of Purified Proteins

Protein structural integrity was assessed by SDS PAGE using the PhastSystem (20% homogeneous gels, GE Healthcare) and by electrospray ionization mass spectrometry (ESI-MS) as detailed previously [10]. CD spectroscopy [44] and native PAGE [10] were carried out as described before.

### Chorismate Mutase Assays

Individual Michaelis-Menten steady-state kinetic parameters were obtained for free MtCM using a continuous assay on a PerkinElmer Lambda 20 spectrophotometer by monitoring initial rates (*v*_0_) of chorismate disappearance at 310 nm (*ε*_310_ = 370 M^−1^ cm^−1^). The substrate (–)-chorismate was prepared from KA12 as previously described [45]. Typically, five chorismate concentrations ([S]) between 100 and ∼2000 µM were tested at 30°C in 50 mM potassium phosphate buffer, pH 7.5. The data were corrected for the background reaction in the absence of a CM at the same temperature and then iteratively fitted to the Michaelis-Menten equation (*v*_0_ = *k*_cat_·[CM]·[S]/(*K*_m_+[S]) by using KaleidaGraph (Synergy Software) yielding *k*_cat_ and *K*_m_ values. The catalytic rate constant *k*_cat_ was calculated per protomer of the dimeric CM.

The *k*_cat_/*K*_m_ values listed in Table 1 were derived from the ratio of individual *k*_cat_ and *K*_m_ parameters each obtained from the fitting of four to six kinetic measurements and stemming from a single set of all MtCM variants and the wild type, prepared and characterized under identical conditions. Independent isolation of the MtCM variants and kinetic assays under slightly variable conditions resulted in non-systematic data fluctuation with an average standard deviation (σ_n-1_) to the values of the homogenous dataset of typically less than 15%. For optimal comparability within the MtCM dataset, we chose to list in Table 1 the calculated *k*_cat_/*K*_m_ values of the homogenous set only, and add a 15% standard deviation to give a conservative estimate of the variation.

*k*_cat_/*K*_m_ estimates for CM activity of the MtCM variants in the presence of an excess of MtDS to ensure complex formation were performed as described previously [10]. Initial velocities (*v*_0 (MtDS+MtCM)_) of a mixture of 30 nM MtCM variant and 300 nM MtDS were obtained by determining the background-corrected decrease of the chorismate absorbance at 274 nm (*ε*_274_ = 2630 M^−1^ cm^−1^). All CM assays with MtDS were carried out in 50 mM BTP (1,3-bis[tris(hydroxymethyl)methylamino]propane), pH 7.5, 0.5 mM TCEP (tris(2-carboxyethyl)phosphine hydrochloride), 0.2 mM phospho*enol*pyruvate, and 0.1 mM MnCl_2_, at a chorismate concentration (range 20–30 µM) below the *K*_m_ of the MtCM-MtDS complex at 30°C. Apparent activation factors were estimated from the ratio of *v*_0 (MtDS+MtCM)_, normalized by MtCM-variant and the spectrometrically determined actual chorismate concentrations in the cuvette, over *k*_cat_/*K*_m_ for the free MtCM variant. Remaining activity of MtCM-MtDS complexes after addition of the feedback inhibitors were determined by dividing *v*_0 (MtDS+MtCM)_ measured in the presence of 100 µM l-Phe and 100 µM l-Tyr by *v*_0 (MtDS+MtCM)_ obtained in the absence of these compounds. The values of Table 1 are the means of two independent determinations, with the standard deviations (σ_n-1_) for the ratios of the individual parameters calculated by error propagation of the experimental σ_n-1_ values.

## Supporting Information

S1 Fig **Nucleotide sequence of plasmid pKTNTET.**
(DOCX)

S2 Fig **Nucleotide sequence of plasmid pT7POLTS.**
(DOCX)

S1 Table **Compilation of amino acids observed in MtCM variants before and after selection experiments (tabular version of** **Fig. 5A–C** **).**
(PDF)
