# Supplementary material for: Functional Mapping of Protein-Protein Interactions in an Enzyme Complex by Directed Evolution
Source: PLoS One. 2014 Dec 31;9(12):e116234. doi: 10.1371/journal.pone.0116234 (PMC4281200; doi:10.1371/journal.pone.0116234)
Supplement: S1 Fig — Nucleotide sequence of plasmid pKTNTET. (DOCX) [file pone.0116234.s001.docx]

**Functional Mapping of Protein-Protein Interactions in an Enzyme Complex by Directed Evolution**

Kathrin Roderer, Martin Neuenschwander, Giosiana Codoni, Severin Sasso, Marianne Gamper and Peter Kast*

**Supporting Figure S1 Nucleotide sequence of plasmid pKTNTET**

1 ACATGCTTAA GACCCACTTT CACATTTAAG TTGTTTTTCT AATCCGCAAA

51 TGATCAATTC AAGGCCGAAT AAGAAGGCTG GCTCTGCACC TTGGTGATCA

101 AATAATTCGA TAGCTTGTCG TAATAATGGC GGCATACTAT CAGTAGTAGG

151 TGTTTCCCTT TCTTCTTTAG CGACTTGATG CTCTTGATCT TCCAATACGC

201 AACCTAAAGT AAAATGCCCC ACAGCGCTGA GTGCATATAA TGCATTCTCT

251 AGTGAAAAAC CTTGTTGGCA TAAAAAGGCT AATTGATTTT CGAGAGTTTC

301 ATACTGTTTT TCTGTAGGCC GTGTACCTAA ATGTACTTTT GCTCCATCGC

351 GATGACTTAG TAAAGCACAT CTAAAACTTT TAGCGTTATT ACGTAAAAAA

401 TCTTGCCAGC TTTCCCCTTC TAAAGGGCAA AAGTGAGTAT GGTGCCTATC

451 TAACATCTCA ATGGCTAAGG CGTCGAGCAA AGCCCGCTTA TTTTTTACAT

501 GCCAATACAA TGTAGGCTGC TCTACACCTA GCTTCTGGGC GAGTTTACGG

551 GTTGTTAAAC CTTCGATTCC GACCTCATTA AGCAGCTCTA ATGCGCTGTT

601 AATCACTTTA CTTTTATCTA ATCTCGACAT CATTAATTCC TAATTTTTGT

651 TGACACTCTA TCATTGATAG AGTTATTTTA CCACTCCCTA TCAGTGATAG

701 AGAAAAGTCT AGCCGATCCC GCGAAATTAA TACGACTCAC TATAGGGAGA

751 CCACAACGGT TTCCCTCTAG AAATAATTTT GTTTAACTTT AAGAAGGAGA

801 TATACATATG CACCATCATC ATCATCATTC TTCTGGTATG CTCGAGTCCC

851 AACCTGTCCC CGAGATCGAC ACGCTGCGCG AAGAGATCGA CCGGCTAGAC

901 GCCGAAATCC TCGCGTTAGT CAAGCGACGC GCTGAGGTTT CCAAGGCCAT

951 CGGCAAGGCC CGGATGGCGT CCGGTGGCAC TCGGCTGGTA CACAGCCGGG

1001 AGATGAAGGT CATCGAACGC TACAGCGAGC TGGGACCCGA CGGTAAGGAT

1051 CTGGCCATCC TGCTTTTGCG ATTGGGCCGT GGCCGCCTCG GTCACTAATA

1101 ACTAGTCAGC TGATCCGGCT GCTAACAAAG CCCGAAAGGA AGCTGAGTTG

1151 GCTGCTGCCA CCGCTGAGCA ATAACTAGCA TAACCCCTTG GGGCCTCTAA

1201 ACGGGTCTTG AGGGGTTTTT TGCTGAAAGG AGGAACTATA TCCGGATTAC

1251 AATTTCAGGT GGCACTTTTC GGGGAAATGT GCGCGGAACC CCTATTTGTT

1301 TATTTTTCTA AATACATTCA AATATGTATC CGCTCATGAG ACAATAACCC

1351 TGATAAATGC TTCAATAATA TTGAAAAAGG AAGAGTATGA GTATTCAACA

1401 TTTCCGTGTC GCCCTTATTC CCTTTTTTGC GGCATTTTGC CTTCCTGTTT

1451 TTGCTCACCC AGAAACGCTG GTGAAAGTAA AAGATGCTGA AGATCAGTTG

1501 GGTGCACGAG TGGGTTACAT CGAACTGGAT CTCAACAGCG GTAAGATCCT

1551 TGAGAGTTTT CGCCCCGAAG AACGTTTTCC AATGATGAGC ACTTTTAAAG

1601 TTCTGCTATG TGGCGCGGTA TTATCCCGTA TTGACGCCGG GCAAGAGCAA

1651 CTCGGTCGCC GCATACACTA TTCTCAGAAT GACTTGGTTG AGTACTCACC

1701 AGTCACAGAA AAGCATCTTA CGGATGGCAT GACAGTAAGA GAATTATGCA

1751 GTGCTGCCAT AACCATGAGT GATAACACTG CGGCCAACTT ACTTCTGACA

1801 ACGATCGGAG GACCGAAGGA GCTAACCGCT TTTTTGCACA ACATGGGGGA

1851 TCATGTAACT CGCCTTGATC GTTGGGAACC GGAGCTGAAT GAAGCCATAC

1901 CAAACGACGA GCGTGACACC ACGATGCCTG TAGCAATGGC AACAACGTTG

1951 CGCAAACTAT TAACTGGCGA ACTACTTACT CTAGCTTCCC GGCAACAATT

2001 AATAGACTGG ATGGAGGCGG ATAAAGTTGC AGGACCACTT CTGCGCTCGG

2051 CCCTTCCGGC TGGCTGGTTT ATTGCTGATA AATCTGGAGC CGGTGAGCGT

2101 GGGTCTCGCG GTATCATTGC AGCACTGGGG CCAGATGGTA AGCCCTCCCG

2151 TATCGTAGTT ATCTACACGA CGGGGAGTCA GGCAACTATG GATGAACGAA

2201 ATAGACAGAT CGCTGAGATA GGTGCCTCAC TGATTAAGCA TTGGTAACTG

2251 TCAGACCAAG TTTACTCATA TATACTTTAG ATTGATTTAA AACTTCATTT

2301 TTAATTTAAA AGGATCTAGG TGAAGATCCT TTTTGATAAT CTCATGACCA

2351 AAATCCCTTA ACGTGAGTTT TCGTTCCACT GAGCGTCAGA CCCCGTAGAA

2401 AAGATCAAAG GATCTTCTTG AGATCCTTTT TTTCTGCGCG TAATCTGCTG

2451 CTTGCAAACA AAAAAACCAC CGCTACCAGC GGTGGTTTGT TTGCCGGATC

2501 AAGAGCTACC AACTCTTTTT CCGAAGGTAA CTGGCTTCAG CAGAGCGCAG

2551 ATACCAAATA CTGTTCTTCT AGTGTAGCCG TAGTTAGGCC ACCACTTCAA

2601 GAACTCTGTA GCACCGCCTA CATACCTCGC TCTGCTAATC CTGTTACCAG

2651 TGGCTGCTGC CAGTGGCGAT AAGTCGTGTC TTACCGGGTT GGACTCAAGA

2701 CGATAGTTAC CGGATAAGGC GCAGCGGTCG GGCTGAACGG GGGGTTCGTG

2751 CACACAGCCC AGCTTGGAGC GAACGACCTA CACCGAACTG AGATACCTAC

2801 AGCGTGAGCT ATGAGAAAGC GCCACGCTTC CCGAAGGGAG AAAGGCGGAC

2851 AGGTATCCGG TAAGCGGCAG GGTCGGAACA GGAGAGCGCA CGAGGGAGCT

2901 TCCAGGGGGA AACGCCTGGT ATCTTTATAG TCCTGTCGGG TTTCGCCACC

2951 TCTGACTTGA GCGTCGATTT TTGTGATGCT CGTCAGGGGG GCGGAGCCTA

3001 TGGAAAAACG CCAGCAACGC GGCCTTTTTA CGGTTCCTGG CCTTTTGCTG

3051 GCCTTTTGCT C
