# Supplementary material for: Functional Mapping of Protein-Protein Interactions in an Enzyme Complex by Directed Evolution
Source: PLoS One. 2014 Dec 31;9(12):e116234. doi: 10.1371/journal.pone.0116234 (PMC4281200; doi:10.1371/journal.pone.0116234)
Supplement: S2 Fig — Nucleotide sequence of plasmid pT7POLTS. (DOCX) [file pone.0116234.s002.docx]

**Functional Mapping of Protein-Protein Interactions in an Enzyme Complex by Directed Evolution**

Kathrin Roderer, Martin Neuenschwander, Giosiana Codoni, Severin Sasso, Marianne Gamper and Peter Kast*

**Supporting Figure S2 Nucleotide sequence of pT7POLTS**

1 GAATTCCGGA TGAGCATTCA TCAGGCGGGC AAGAATGTGA ATAAAGGCCG

51 GATAAAACTT GTGCTTATTT TTCTTTACGG TCTTTAAAAA GGCCGTAATA

101 TCCAGCTGAA CGGTCTGGTT ATAGGTACAT TGAGCAACTG ACTGAAATGC

151 CTCAAAATGT TCTTTACGAT GCCATTGGGA TATATCAACG GTGGTATATC

201 CAGTGATTTT TTTCTCCATT TTAGCTTCCT TAGCTCCTGA AAATCTCGAT

251 AACTCAAAAA ATACGCCCGG TAGTGATCTT ATTTCATTAT GGTGAAAGTT

301 GGAACCTCTT ACGTGCCGAT CAACGTCTCA TTTTCGCCAA AAGTTGGCCC

351 AGGGCTTCCC GGTATCAACA GGGACACCAG GATTTATTTA TTCTGCGAAG

401 TGATCTTCCG TCACAGGTAT TTATTCGGCG CAAAGTGCGT CGGGTGATGC

451 TGCCAACTTA CTGATTTAGT GTATGATGGT GTTTTTGAGG TGCTCCAGTG

501 GCTTCTGTTT CTATCAGCTG TCCCTCCTGT TCAGCTACTG ACGGGGTGGT

551 GCGTAACGGC AAAAGCACCG CCGGACATCA GCGCTAGCGG AGTGTATACT

601 GGCTTACTAT GTTGGCACTG ATGAGGGTGT CAGTGAAGTG CTTCATGTGG

651 CAGGAGAAAA AAGGCTGCAC CGGTGCGTCA GCAGAATATG TGATACAGGA

701 TATATTCCGC TTCCTCGCTC ACTGACTCGC TACGCTCGGT CGTTCGACTG

751 CGGCGAGCGG AAATGGCTTA CGAACGGGGC GGAGATTTCC TGGAAGATGC

801 CAGGAAGATA CTTAACAGGG AAGTGAGAGG GCCGCGGCAA AGCCGTTTTT

851 CCATAGGCTC CGCCCCCCTG ACAAGCATCA CGAAATCTGA CGCTCAAATC

901 AGTGGTGGCG AAACCCGACA GGACTATAAA GATACCAGGC GTTTCCCCCT

951 GGCGGCTCCC TCGTGCGCTC TCCTGTTCCT GCCTTTCGGT TTACCGGTGT

1001 CATTCCGCTG TTATGGCCGC GTTTGTCTCA TTCCACGCCT GACACTCAGT

1051 TCCGGGTAGG CAGTTCGCTC CAAGCTGGAC TGTATGCACG AACCCCCCGT

1101 TCAGTCCGAC CGCTGCGCCT TATCCGGTAA CTATCGTCTT GAGTCCAACC

1151 CGGAAAGACA TGCAAAAGCA CCACTGGCAG CAGCCACTGG TAATTGATTT

1201 AGAGGAGTTA GTCTTGAAGT CATGCGCCGG TTAAGGCTAA ACTGAAAGGA

1251 CAAGTTTTGG TGACTGCGCT CCTCCAAGCC AGTTACCTCG GTTCAAAGAG

1301 TTGGTAGCTC AGAGAACCTT CGAAAAACCG CCCTGCAAGG CGGTTTTTTC

1351 GTTTTCAGAG CAAGAGATTA CGCGCAGACC AAAACGATCT CAAGAAGATC

1401 ATCTTATTAA TCAGATAAAA TATTTCTAGC TAGATTTCAG TGCAATTTAT

1451 CTCTTCAAAT GTAGCACCTG AAGTCAGCCC CATACGATAT AAGTTGTAAT

1501 TCTCATGTTT GACAGCTTAT CATCGATAAG CTTGCATGCT TAAGACCCAC

1551 TTTCACATTT AAGTTGTTTT TCTAATCCGC AAATGATCAA TTCAAGGCCG

1601 AATAAGAAGG CTGGCTCTGC ACCTTGGTGA TCAAATAATT CGATAGCTTG

1651 TCGTAATAAT GGCGGCATAC TATCAGTAGT AGGTGTTTCC CTTTCTTCTT

1701 TAGCGACTTG ATGCTCTTGA TCTTCCAATA CGCAACCTAA AGTAAAATGC

1751 CCCACAGCGC TGAGTGCATA TAATGCATTC TCTAGTGAAA AACCTTGTTG

1801 GCATAAAAAG GCTAATTGAT TTTCGAGAGT TTCATACTGT TTTTCTGTAG

1851 GCCGTGTACC TAAATGTACT TTTGCTCCAT CGCGATGACT TAGTAAAGCA

1901 CATCTAAAAC TTTTAGCGTT ATTACGTAAA AAATCTTGCC AGCTTTCCCC

1951 TTCTAAAGGG CAAAAGTGAG TATGGTGCCT ATCTAACATC TCAATGGCTA

2001 AGGCGTCGAG CAAAGCCCGC TTATTTTTTA CATGCCAATA CAATGTAGGC

2051 TGCTCTACAC CTAGCTTCTG GGCGAGTTTA CGGGTTGTTA AACCTTCGAT

2101 TCCGACCTCA TTAAGCAGCT CTAATGCGCT GTTAATCACT TTACTTTTAT

2151 CTAATCTCGA CATCATTAAT TCCTAATTTT TGTTGACACT CTATCATTGA

2201 TAGAGTTATT TTACCACTCC CTATCAGTGA TAGAGAAAAG TCTAGAGGAA

2251 GAGGCACTAA ATGAACACGA TTAACATCGC TAAGAACGAC TTCTCTGACA

2301 TCGAACTGGC TGCTATCCCG TTCAACACTC TGGCTGACCA TTACGGTGAG

2351 CGTTTAGCTC GCGAACAGTT GGCCCTTGAG CATGAGTCTT ACGAGATGGG

2401 TGAAGCACGC TTCCGCAAGA TGTTTGAGCG TCAACTTAAA GCTGGTGAGG

2451 TTGCGGATAA CGCTGCCGCC AAGCCTCTCA TCACTACCCT ACTCCCTAAG

2501 ATGATTGCAC GCATCAACGA CTGGTTTGAG GAAGTGAAAG CTAAGCGCGG

2551 CAAGCGCCCG ACAGCCTTCC AGTTCCTGCA AGAAATCAAG CCGGAAGCCG

2601 TAGCGTACAT CACCATTAAG ACCACTCTGG CTTGCCTAAC CAGTGCTGAC

2651 AATACAACCG TTCAGGCTGT AGCAAGCGCA ATCGGTCGGG CCATTGAGGA

2701 CGAGGCTCGC TTCGGTCGTA TCCGTGACCT TGAAGCTAAG CACTTCAAGA

2751 AAAACGTTGA GGAACAACTC AACAAGCGCG TAGGGCACGT CTACAAGAAA

2801 GCATTTATGC AAGTTGTCGA GGCTGACATG CTCTCTAAGG GTCTACTCGG

2851 TGGCGAGGCG TGGTCTTCGT GGCATAAGGA AGACTCTATT CATGTAGGAG

2901 TACGCTGCAT CGAGATGCTC ATTGAGTCAA CCGGAATGGT TAGCTTACAC

2951 CGCCAAAATG CTGGCGTAGT AGGTCAAGAC TCTGAGACTA TCGAACTCGC

3001 ACCTGAATAC GCTGAGGCTA TCGCAACCCG TGCAGGTGCG CTGGCTGGCA

3051 TCTCTCCGAT GTTCCAACCT TGCGTAGTTC CTCCTAAGCC GTGGACTGGC

3101 ATTACTGGTG GTGGCTATTG GGCTAACGGT CGTCGTCCTC TGGCGCTGGT

3151 GCGTACTCAC AGTAAGAAAG CACTGATGCG CTACGAAGAC GTTTACATGC

3201 CTGAGGTGTA CAAAGCGATT AACATTGCGC AAAACACCGC ATGGAAAATC

3251 AACAAGAAAG TCCTAGCGGT CGCCAACGTA ATCACCAAGT GGAAGCATTG

3301 TCCGGTCGAG GACATCCCTG CGATTGAGCG TGAAGAACTC CCGATGAAAC

3351 CGGAAGACAT CGACATGAAT CCTGAGGCTC TCACCGCGTG GAAACGTGCT

3401 GCCGCTGCTG TGTACCGCAA GGACAAGGCT CGCAAGTCTC GCCGTATCAG

3451 CCTTGAGTTC ATGCTTGAGC AAGCCAATAA GTTTGCTAAC CATAAGGCCA

3501 TCTGGTTCCC TTACAACATG GACTGGCGCG GTCGTGTTTA CGCTGTGTCA

3551 ATGTTCAACC CGCAAGGTAA CGATATGACC AAAGGACTGC TTACGCTGGC

3601 GAAAGGTAAA CCAATCGGTA AGGAAGGTTA CTACTGGCTG AAAATCCACG

3651 GTGCAAACTG TGCGGGTGTC GATAAGGTTC CGTTCCCTGA GCGCATCAAG

3701 TTCATTGAGG AAAACCACGA GAACATCATG GCTTGCGCTA AGTCTCCACT

3751 GGAGAACACT TGGTGGGCTG AGCAAGATTC TCCGTTCTGC TTCCTTGCGT

3801 TCTGCTTTGA GTACGCTGGG GTACAGCACC ACGGCCTGAG CTATAACTGC

3851 TCCCTTCCGC TGGCGTTTGA CGGGTCTTGC TCTGGCATCC AGCACTTCTC

3901 CGCGATGCTC CGAGATGAGG TAGGTGGTCG CGCGGTTAAC TTGCTTCCTA

3951 GTGAAACCGT TCAGGACATC TACGGGATTG TTGCTAAGAA AGTCAACGAG

4001 ATTCTACAAG CAGACGCAAT CAATGGGACC GATAACGAAG TAGTTACCGT

4051 GACCGATGAG AACACTGGTG AAATCTCTGA GAAAGTCAAG CTGGGCACTA

4101 AGGCACTGGC TGGTCAATGG CTGGCTTACG GTGTTACTCG CAGTGTGACT

4151 AAGCGTTCAG TCATGACGCT GGCTTACGGG TCCAAAGAGT TCGGCTTCCG

4201 TCAACAAGTG CTGGAAGATA CCATTCAGCC AGCTATTGAT TCCGGCAAGG

4251 GTCTGATGTT CACTCAGCCG AATCAGGCTG CTGGATACAT GGCTAAGCTG

4301 ATTTGGGAAT CTGTGAGCGT GACGGTGGTA GCTGCGGTTG AAGCAATGAA

4351 CTGGCTTAAG TCTGCTGCTA AGCTGCTGGC TGCTGAGGTC AAAGATAAGA

4401 AGACTGGAGA GATTCTTCGC AAGCGTTGCG CTGTGCATTG GGTAACTCCT

4451 GATGGTTTCC CTGTGTGGCA GGAATACAAG AAGCCTATTC AGACGCGCTT

4501 GAACCTGATG TTCCTCGGTC AGTTCCGCTT ACAGCCTACC ATTAACACCA

4551 ACAAAGATAG CGAGATTGAT GCACACAAAC AGGAGTCTGG TATCGCTCCT

4601 AACTTTGTAC ACAGCCAAGA CGGTAGCCAC CTTCGTAAGA CTGTAGTGTG

4651 GGCACACGAG AAGTACGGAA TCGAATCTTT TGCACTGATT CACGACTCCT

4701 TCGGTACCAT TCCGGCTGAC GCTGCGAACC TGTTCAAAGC AGTGCGCGAA

4751 ACTATGGTTG ACACATATGA GTCTTGTGAT GTACTGGCTG ATTTCTACGA

4801 CCAGTTCGCT GACCAGTTGC ACGAGTCTCA ATTGGACAAA ATGCCAGCAC

4851 TTCCGGCTAA AGGTAACTTG AACCTCCGTG ACATCTTAGA GTCGGACTTC

4901 GCGTTCGCGC TCGAGGCGGC GAACGATGAA AACTATGCGC TGGCGGCGTA

4951 ATCTAGTCAG CTGATCCGGC TGCTAACAAA GCCCGAAAGG AAGCTGAGTT

5001 GGCTGCTGCC ACCGCTGAGC AATAACTAGC ATAACCCCTT GGGGCCTCTA

5051 AACGGGTCTT GAGGGGTTTT TTGCTGAAAG GAGGAACTAT ATCCGGGGAT

5101 CTGCATCGCA GGATGCTGCT GGCTACCCTG TGGAACACCT ACATCTGTAT

5151 TAACGAAGCG CTAACCGTTT TTATCAGGCT CTGGGAGGCA GAATAAATGA

5201 TCATATCGTC AATTATTACC TCCACGGGGA GAGCCTGAGC AAACTGGCCT

5251 CAGGCATTTG AGAAGCACAC GGTCACACTG CTTCCGGTAG TCAATAAACC

5301 GGTAAACCAG CAATAGACAT AAGCGGCTAT TTAACGACCC TGCCCTGAAC

5351 CGACGACCGG GTCGAATTTG CTTTCGAATT TCTGCCATTC ATCCGCTTAT

5401 TATCACTTAT TCAGGCGTAG CACCAGGCGT TTAAGGGCAC CAATAACTGC

5451 CTTAAAAAAA TTACGCCCCG CCCTGCCACT CATCGCAGTA CTGTTGTAAT

5501 TCATTAAGCA TTCTGCCGAC ATGGAAGCCA TCACAGACGG CATGATGAAC

5551 CTGAATCGCC AGCGGCATCA GCACCTTGTC GCCTTGCGTA TAATATTTGC

5601 CCATGGTGAA AACGGGGGCG AAGAAGTTGT CCATATTGGC CACGTTTAAA

5651 TCAAAACTGG TGAAACTCAC CCAGGGATTG GCTGAGACGA AAAACATATT

5701 CTCAATAAAC CCTTTAGGGA AATAGGCCAG GTTTTCACCG TAACACGCCA

5751 CATCTTGCGA ATATATGTGT AGAAACTGCC GGAAATCGTC GTGGTATTCA

5801 CTCCAGAGCG ATGAAAACGT TTCAGTTTGC TCATGGAAAA CGGTGTAACA

5851 AGGGTGAACA CTATCCCATA TCACCAGCTC ACCGTCTTTC ATTGCCATAC

5901 G
