# Supplementary material for: Functional Mapping of Protein-Protein Interactions in an Enzyme Complex by Directed Evolution
Source: PLoS One. 2014 Dec 31;9(12):e116234. doi: 10.1371/journal.pone.0116234 (PMC4281200; doi:10.1371/journal.pone.0116234)
Supplement: S1 Table — Compilation of amino acids observed in MtCM variants before and after selection experiments (tabular version of Fig. 5A–C ). (PDF) [file pone.0116234.s003.pdf]

Functional Mapping of Protein-Protein Interactions in an Enzyme Complex by Directed Evolution

Kathrin Roderer, Martin Neuenschwander, Giosiana Codoni, Severin Sasso, Marianne Gamper and Peter Kast*

Supporting Table S1 Compilation of amino acids observed in MtCM variants before and after selection (Fig. 5A-C tabular version)

(A) Amino acid residues found under non-selective conditions (M9c +FY)

Wt	Position G84		Position R85		Position G86		Position R87		Position L88		Position G89		Position H90	
Residue	in %	found / library size	in %	found / library size	in %	found / library size	in %	found / library size	in %	found / library size	in %	found / library size	in %	found / library size
G			5	2/41	6	2/35	5	2/39			18	6/34	9	3/34
A	19	6/32			3	1/35	8	3/39	3	1/31	6	2/34	9	3/34
S	13	4/32	5	2/41	9	3/35	10	4/39	10	3/31	15	5/34	12	4/34
C	6	2/32	5	2/41			3	1/39	3	1/31			3	1/34
T			15	6/41	6	2/35	5	2/39	13	4/31	9	3/34	3	1/34
D	6	2/32	12	5/41	6	2/35	5	2/39	6	2/31	9	3/34		
P	6	2/32	2	1/41	11	4/35	5	2/39	6	2/31			3	1/34
N			7	3/41	6	2/35								
V			5	2/41	9	3/35	10	4/39	6	2/31	6	2/34	3	1/34
E					3	1/35	5	2/39			3	1/34	3	1/34
Q			2	1/41			10	4/39	6	2/31	3	1/34		
H	3	1/32	2	1/41	6	2/35	3	1/39					15	5/34
L	13	4/32	10	4/41	3	1/35	8	3/39	6	2/31	9	3/34	6	2/34
I	6	2/32	7	3/41	14	5/35	3	1/39	10	3/31	9	3/34	12	4/34
M					3	1/35	3	1/39	10	3/31			3	1/34
K			2	1/41	6	2/35					3	1/34		
R	13	4/32	7	3/41			5	2/39	6	2/31	3	1/34	15	5/34
F	3	1/32	7	3/41	6	2/35	5	2/39	3	1/31	3	1/34	3	1/34
Y	6	2/32			3	1/35	5	2/39	3	1/31	3	1/34		
W			2	1/41										
*	6	2/32	2	1/41	3	1/35	3	1/39	6	2/31	3	1/34	3	1/34

(B) Preferred residues selected under a stringent regime (M9c, no inducer present) in the presence of MtDS (pKIMP-ACG)

Wt	Position G84		Position R85		Position G86		Position R87		Position L88		Position G89		Position H90	
Residue	in %	found / library size	in %	found / library size	in %	found / library size	in %	found / library size	in %	found / library size	in %	found / library size	in %	found / library size
G	73	33/45			100	64/64	1	1/89			51	40/78	23	13/57
A	18	8/45					10	9/89			3	2/78	4	2/57
S	4	2/45					12	11/89	3	2/66	10	8/78	7	4/57
C									2	1/66			2	1/57
T	4	2/45					8	7/89	2	1/66			11	6/57
D														
P							7	6/89	11	7/66			2	1/57
N							8	7/89			1	1/78		
V							4	4/89	5	3/66	3	2/78	4	2/57
E							1	1/89					2	1/57
Q							3	3/89					5	3/57
H							4	4/89					2	1/57
L							10	9/89	62	41/66			2	1/57
I							1	1/89	8	5/66			2	1/57
M									9	6/66				
K							9	8/89					2	1/57
R			100	83/83			19	17/89			6	5/78	21	12/57
F													11	6/57
Y														
W							1	1/89					2	1/57
*											26	20/78	2	1/57

(C) Residue patterns emerging in selected MtCM variants grown in the absence of MtDS (pKIMP-UAUC)

Wt	Position G84		Position R85		Position G86		Position R87		Position L88		Position G89		Position H90	
Residue	in %	found / library size	in %	found / library size	in %	found / library size	in %	found / library size	in %	found / library size	in %	found / library size	in %	found / library size
G	17	11/63			70	44/63	2	1/62	8	5/65	3	2/63	12	7/60
A			2	1/63			8	5/62	5	3/65	5	3/63	5	3/60
S	43	27/63			3	2/63	11	7/62	14	9/65	5	3/63	5	3/60
C	35	22/63	3	2/63			3	2/62	5	3/65	3	2/63		
T			10	6/63	6	4/63	6	4/62	8	5/65	5	3/63	12	7/60
D							8	5/62			3	2/63	7	4/60
P							15	9/62	3	2/65	21	13/63	8	5/60
N									5	3/65	3	2/63	3	2/60
V			2	1/63	2	1/63	3	2/62	12	8/65	2	1/63	7	4/60
E							10	6/62	2	1/65	5	3/63		
Q			3	2/63			3	2/62	5	3/65	3	2/63	2	1/60
H							2	1/62	8	5/65	3	2/63	2	1/60
L					5	3/63	6	4/62	11	7/65	8	5/63	10	6/60
I							5	3/62	3	2/65	5	3/63		
M	3	2/63									2	1/63		
K			3	2/63	2	1/63	3	2/62	5	3/65	5	3/63	3	2/60
R			76	48/63	10	6/63	11	7/62	6	4/65	6	4/63	3	2/60
F	2	1/63									5	3/63	2	1/60
Y			2	1/63							5	3/63	3	2/60
W													7	4/60
*					3	2/63	3	2/62	3	2/65	5	3/63	10	6/60
